# Anthelmintic flavonoids and other com­pounds from *Combretum glutinosum* Perr. ex DC (Combretaceae) leaves

**DOI:** 10.1107/S2053229621007841

**Published:** 2021-08-06

**Authors:** Placide M. Toklo, Eléonore Yayi Ladekan, Anthony Linden, Sylvie Hounzangbe-Adote, Siméon F. Kouam, Joachim D. Gbenou

**Affiliations:** a Laboratoire de Pharmacognosie et des Huiles Essentielles, Facultés des Sciences et Techniques, Université d’Abomey Calavi, 01 BP: 918 ISBA Cotonou, Benin; b Department of Chemistry, University of Zurich, Winterthurerstrasse 190, CH-8057 Zurich, Switzerland; c Laboratoire d’Ethnopharmacologie et de Santé Animale, Faculté des Sciences Agronomiques, Université d’Abomey Calavi, 01 BP: 526 Cotonou, Benin; d Department of Chemistry, Higher Teacher Training College, University of Yaounde I, PO Box 47, 4124 Yaounde, Cameroon

**Keywords:** *C. glutinosum*, flavonoids, triterpenes, crystal structure, anthelmintic, natural product, absolute configuration

## Abstract

A chemical study of the hydro-ethanol extract of the leaves of *Combretum glutinosum* resulted in the isolation of nine com­pounds, including 5-de­methyl­sin­en­se­tin (**1**), umuhengerin (**2**), (20*S*,24*R*)-ocotillone (**3**), lupeol (**4**), β-sitosterol (**5**), oleanolic acid (**6**), betulinic acid (**7**), corymbosin (**8**) and β-sito­sterol glucoside (**9**). Four com­pounds have been isolated for the first time from the genus *Combretum* [*viz.* (**1**), (**2**), (**3**) and (**8**)]. The crystal structures of flavonoid (**2**), C_20_H_20_O_8_, *Z*′ = 2, and triterpene (**3**), C_30_H_50_O_3_, *Z*′ = 1, have been determined for the first time; the latter confirmed the absolute configuration of native (20*S*,24*R*)-ocotillone previously derived from the crystal structures of related derivatives. The mol­ecules of (**3**) are linked into supra­molecular chains by inter­molecular O—H⋯O hydrogen bonds. The crude extracts obtained by aqueous decoction and hydro-ethano­lic maceration, as well as the nine isolated com­pounds, were tested for their anthelmintic activity on the larvae and adult worms of *Haemonchus contortus*, a hematophage that causes parasitic disorders in small ruminants. The evaluated anthelmintic activity showed that the extracts at different doses, as well as all the com­pounds tested at 150 µg ml^−1^, inhibited the migration of the larvae and the motility of the adult worms of the parasite com­pared with the phosphate buffer solution negative reference control. The best activity was obtained with flavonoids (**1**), (**2**) and (**8**) on both stages of the parasite. The flavones that showed good activity can be used for the further development of other derivatives, which could increase the anthelmintic efficacy.

## Abstract 2

Nine known com­pounds have been isolated from *Combretum glutinosum* leaves, including flavonoids and triterpenes. The crystal structures of umuhengerin and (20*S*,24*R*)-ocotillone are reported for the first time; the latter structure confirms the absolute configuration. The crude extracts and the isolated com­pounds were tested for their anthelmintic activity on the larvae and adult worms of *Haemonchus contortus*. The best activity on both stages of the parasite was obtained with the flavonoids.

## Introduction   

Combretaceae are trees, shrubs or often lianas widely distributed in subtropical to tropical regions. This family consists of 18 genera, including 370 species of *Combretum* (Malgras, 1992 ▸; McGaw *et al.*, 2001 ▸, Amadou, 2004 ▸). These species are widely used in traditional medicine for their numerous pharmacological properties (Komlan, 2002 ▸). *C. glutinosum* is a tree of the genus *Combretum* belonging to the family Combretaceae. This plant is most often present in tree savannas, normally on shallow soils (Akoègninou *et al.*, 2006 ▸). It is distributed in tropical Africa from Mauritania to Uganda, passing through, for example, Senegal, Cameroon and Chad. In Bénin, the plant is spread in the North in Kandi, Kétou, Toukountouna, south of Malanville, Bessassi and Porga, and in the Pendrari Park (Akoègninou *et al.*, 2006 ▸). This species is among the most widely used of the medicinal plants in West Africa (Kerharo & Adam, 1974 ▸). It has been reported by Toklo *et al.* (2021 ▸) that it is used in the treatment of malaria, dysentery, diarrhea, bronchitis and hypertension. The traditional uses of this plant have led to numerous pharmacological studies, including anti­bacterial, anti­fungal, anthelmintic, anti­malarial and anti­drepanosite properties (Baba-Moussa *et al.*, 1999 ▸; Ouattara *et al.*, 2006 ▸; Usman *et al.*, 2017 ▸; Sall *et al.*, 2017 ▸; Alowanou *et al.*, 2019 ▸). Previous phytochemical studies of the genus *Combretum* led to the isolation of tannins, flavonoids, triterpenoids and steroids (Jossang *et al.*, 1994 ▸; Dawe *et al.*, 2013 ▸; Roy *et al.*, 2014 ▸, Amako *et al.*, 2016 ▸; Sene *et al.*, 2018 ▸; N’Diaye *et al.*, 2017 ▸; Balde *et al.*, 2019 ▸). In the search for a new active ingredient effective against increasing biological resistance to synthetic anthelmintics, the study reported here was undertaken on the leaves of *C. glutinosum*, which were obtained from plants in Bénin. The search for bioactive secondary metabolites from the leaves revealed nine known com­pounds (Scheme 1), of which the crystal structures of two, one flavonoid and one triterpene, have been determined for the first time. The biological activity of these com­pounds on the larvae and adult worms of *H. contortus*, a hematophage that causes parasitic disorders in small ruminants, has also been investigated.

## Experimental   

### Chromatographic and spectroscopic analysis   

Column chromatography was performed using 230–400 mesh silica gel (Merck, Darmstadt, Germany), 70–230 mesh silica gel (Merck) and sephadex LH-20 (Sigma–Aldrich). Thin-layer chromatography (TLC) was performed on a pre-coated aluminium sheet of silica gel 60 F254 (Merck). The spots of com­pounds were detected using UV lamps at two wavelengths (254 and 365 nm) and then fixed using a 10% sulfuric acid spray reagent, followed by heating to 373 K. The high-resolution mass spectra were recorded in positive mode using a QTOF mass spectrometer (Bruker, Germany) equip­ped with an HESI source. The spectrometer operates in posi­tive mode (mass range 100–1500, with a scan rate of 1.00 Hz), with automatic gain control to provide high accuracy mass measurements within the mass range. NMR spectra were recorded in deuterated chloro­form (CDCl_3_) and/or deuterated methanol (MeOD) using a Bruker DRX 500 NMR spec­trom­eter (Bruker, Rheinstetten, Germany); the chemical shifts (δ) are given in ppm relative to tetra­methyl­silane (TMS) (Sigma–Aldrich, Germany) as the inter­nal standard.

### Collection of plant material, extraction and isolation of compounds   

The leaves of *C. glutinosum* were collected in April 2018 in Kandi (in northern Bénin) and identified at the national herbarium of the University of Abomey–Calavi. A reference specimen was stored under the accession number YH 241/HNB after authentication.

The leaves were dried in the shade for two weeks before pulverization. The leaf powder (500 g) was macerated three times in 10 l of an ethanol/water (7:3 *v*/*v*) mixture at room temperature for 72 h. After filtration, the crude extract (67 g) was obtained by evaporation of the solvent under reduced pressure using a rotary evaporator equipped with a vacuum pump. Different systems were used for TLC of the extract in order to find the best separation system. The extract was separated directly by silica-gel column chromatography. The column was eluted with mixtures of hexa­ne–ethyl acetate (hex/EtOAc) and methanol with increasing polarity to give 92 fractions of 200 ml each. They were grouped on the basis of their TLC profile into five main fractions, *i.e.* FCG1 (Hex/EtOAc 10%, 5.3 g), FCG2 (Hex/EtOAc 20%, 12.9 g), FCG3 (Hex/EtOAc 30%, 5.6 g), FCG4 (Hex/EtOAc 40–50%, 5 g) and FCG5 (MeOH, 21.6 g), with one pure com­pound, lupeol [(**4**); 13 mg], obtained in the hex/EtOAc 10% system.
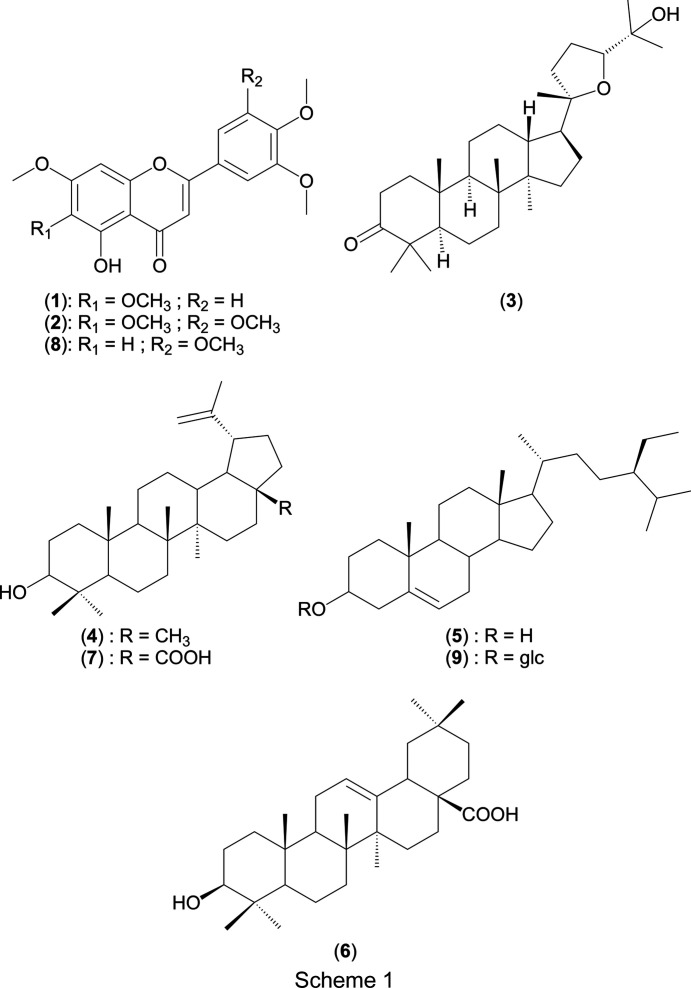


The FCG2 fraction was purified by silica-gel column chromatography using an isocratic system of hex/EtOAc (17:3 *v*/*v*) to give betulinic acid [(**7**); 35 mg], oleanolic acid [(**6**); 12 mg], β-sitosterol [(**5**); 26 mg], and (20*S*,24*R*)-ocotillone [(**3**); 55 mg], as well as two subfractions, FCG2-1 and FCG2-2. The FCG2-1 subfraction (2.1 g) was separated on a Sephadex LH-20 column by eluting with di­chloro­methane–methanol (4:6 *v*/*v*) to yield corymbosin [(**8**); 6 mg].

Based on the TLC profiles, the FCG2-2 subfraction was combined with the FCG3 fraction and subjected to silica-gel column chromatography using a gradient elution of hex/EtOAc with increasing polarity to obtain the com­pounds 5-de­­methyl­sinensetin [(**1**); 17 mg] and umuhengerin [(**2**) 22 mg]. The FCG4 fraction was also eluted with a mixture of ethyl acetate and 5% methanol to give eight subfractions (FCG4 1–8), which all contained an impure com­pound (CCG20). The FCG4-2 fraction was passed through a Sepha­dex LH-20 column and eluted with methanol to give solely pure CCG20, which was identified as β-sitosterol glucoside [(**9**); 48 mg].

Colourless needle-like crystals of (**2**) and colourless plate-like crystals of (**3**) were obtained by slow diffusion of di­chloro­methane into their solutions in methanol. Selected crystals were mounted on cryo loops.

### Aqueous extract   

An aqueous extract was obtained by boiling 100 g of *C. glutinosum* leaf powder in 1000 ml of distilled water brought to the boil for 30 min. After deca­ntation, the mixture was filtered on Whatman paper and the filtrate obtained was evaporated under vacuum to obtain the dry extract.

### Anthelmintic tests   

#### Test for inhibition of larval migration and motility of adult worms   

The test of larval migration and motility of adult worms in the presence of the samples was evaluated following the procedure of Hounzangbe-Adote *et al.* (2005 ▸). The observation of the worms in the presence of the extracts was done every 6 h and every 3 h in the presence of the com­pounds. The concentration of the tested com­pounds was 150 µg ml^−1^ in phosphate buffer solution (PBS, pH 7 and 0.15 *M*), analogous to that used by Brunet & Hoste (2006 ▸). Levamisole and PBS were used as positive and negative reference controls, respectively.

#### Statistical analysis   

The different values were included in a two-criteria repeated measures analysis of variance model. The com­parison of means for the different tests was done using the SNK procedure, which runs the Student–Newman–Keuls test in the R software. Differences were considered significant at the 5% level.

### Refinement   

Crystal data, data collection and structure refinement details for (**2**) and (**3**) are summarized in Table 1 ▸. For both structures, the hy­droxy H atoms were located in a difference Fourier map and their positions were refined freely along with individual isotropic displacement parameters. The methyl H atoms were constrained to an ideal geometry (C—H = 0.98 Å), with *U*_iso_(H) = 1.5*U*_eq_(C), but were allowed to rotate freely about the C—C bonds. All other H atoms were placed in geometrically idealized positions and constrained to ride on their parent atoms, with C—H distances of 0.95 (aromatic), 0.99 (methyl­ene) or 1.00 Å (methine) and with *U*_iso_(H) = 1.2*U*_eq_(C). The absolute configuration of (**3**) was determined confidently from the diffraction experiment by refinement of the absolute structure parameter using the intensity quotients method (Parsons *et al.*, 2013 ▸). For (**2**), one reflection was omitted from the final cycles of refinement because its observed intensity was much lower than the calculated value as a result of being partially obscured by the beam stop; a correction for secondary extinction was also applied.

## Results and discussion   

### Identification of com­pounds   

Repeated column chromatography of *C. glutinosum* leaf hydro-ethanol extract followed by silica-gel and sephadex LH-20 column purification yielded nine known com­pounds: 5-de­methyl­sinensetin, (**1**) (Khazneh *et al.*, 2016 ▸), umuhengerin, (**2**) (Rwangabo *et al.*, 1988 ▸; Imbenzi *et al.*, 2014 ▸), (20*S*,24*R*)-ocotillone, (**3**) (Aalbersberg *et al.*, 1991 ▸), lupeol, (**4**) (Sholichin *et al.*, 1980 ▸; Banskota *et al.*, 2000 ▸; Balde *et al.*, 2019 ▸), β-sitosterol, (**5**) (Rubinstein *et al.*, 1976 ▸; Banskota *et al.*, 2000 ▸), oleanolic acid, (**6**) (Mahato & Kundu, 1994 ▸), betulinic acid, (**7**) (Sholichin *et al.*, 1980 ▸; Banskota *et al.*, 2000 ▸), corymbosin, (**8**) (Çitoğlu *et al.*, 2003 ▸), and β-sitosterol glucoside, (**9**) (Adnyana *et al.*, 2000 ▸) (Scheme 1). The structures of the com­pounds were established by inter­pretation of their spectroscopic data, mainly 1D NMR [^1^H, ^13^C and DEPT (distortionless enhancement by polarization transfer)], 2D NMR [COSY (correlated spectroscopy), HSQC (heteronuclear single quantum coherence) and HMBC (heteronuclear multiple bond correlation)] and mass spectrometry, and by com­parison with literature data. Although all of these com­pounds are known, com­pounds (**1**), (**2**), (**3**) and (**8**) have been isolated for the first time from the genus *Combretum* and the crystal structures of com­pounds (**2**) and (**3**), previously undetermined, have been established.

### The crystal structures of (2) and (3)   

**Figure 1:**
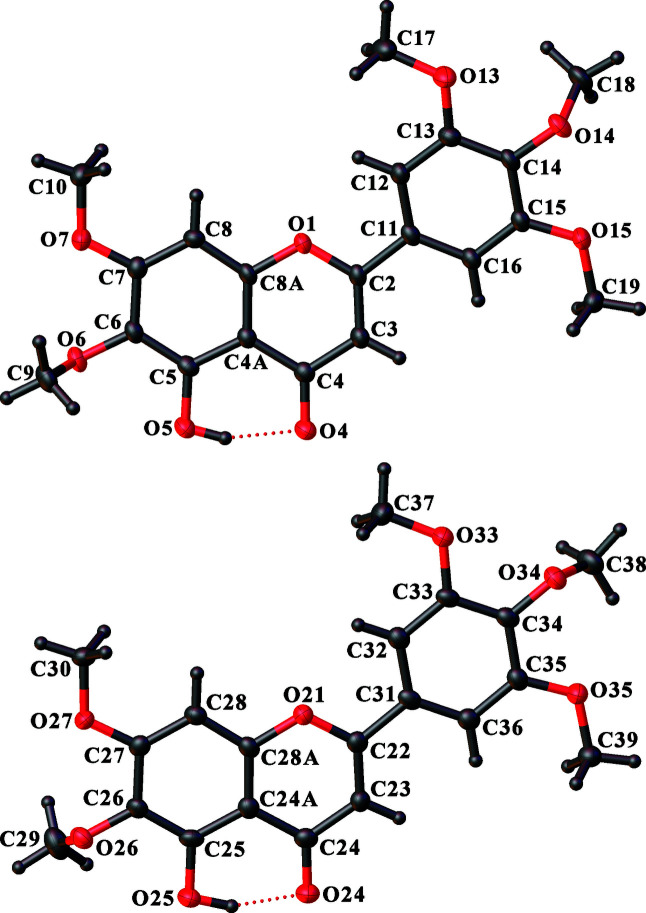
Separate views of the two symmetry-independent mol­ecules of (**2**), showing the atom-labelling scheme. Displacement ellipsoids are drawn at the 50% probability level. H atoms are represented by spheres of arbitrary size.

The flavonoid umuhengerin, (**2**), was originally isolated from the leaves of *Lantana trifolia* L. (Verbenaceae) and found to display *in vitro* anti­bacterial and anti­fungal properties (Rwangabo *et al.*, 1988 ▸). In the crystal structure of (**2**), there are two symmetry-independent mol­ecules in the asymmetric unit (Fig. 1 ▸). The conformations of these mol­ecules differ primarily in the orientations of the C6/C26 and C14/C34 meth­oxy groups, which are the substituents adjacent to the hy­droxy group and at the 4-position of the tri­meth­oxy­phenyl ring, respectively. In the former case, these meth­oxy C—O torsion angles differ by 15.7 (3)°, while the rotation is 164.81 (3)° in the latter case (calculated when one mol­ecule is overlaid with the inverted form of the other mol­ecule, as allowed by the space-group symmetry). Apart from the methyl groups of these meth­oxy substituents, both flavonoid mol­ecules are essentially planar, with r.m.s. deviations of all ring C and O atoms of 0.27 and 0.14 Å for the mol­ecules containing atoms O1 and O21, respectively, although there may be a little bowing along the axis of the three-ring system. The dihedral angles between the individual planes of the phenyl and fused rings are 7.18 (8) and 3.05 (8)°, respectively.

**Figure 2:**
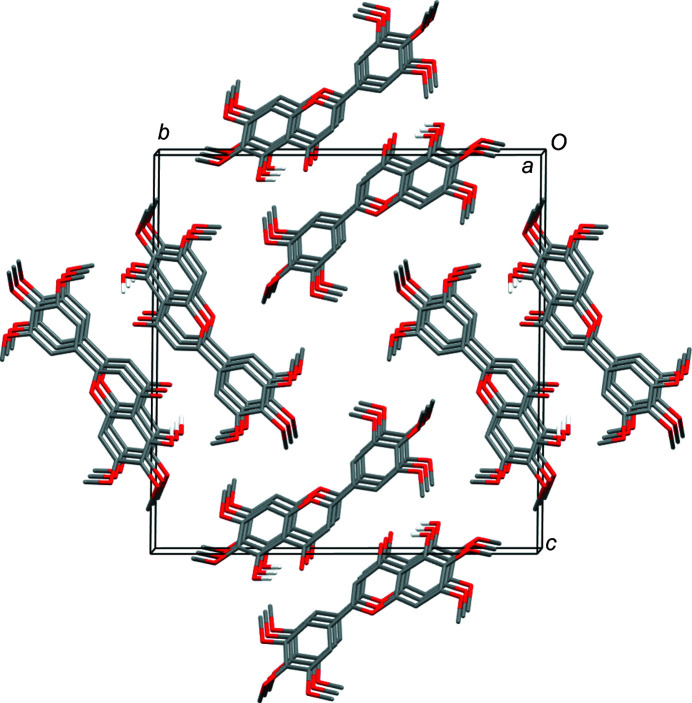
The crystal packing of (**2**), viewed down the *a* axis, showing the centrosymmetric double-stack columns of mol­ecules, with the columns at the top and bottom being com­posed solely of one of the symmetry-independent types of mol­ecules and the columns on the left and right being com­posed solely of the other independent type.

The hy­droxy group in each independent flavonoid mol­ecule forms an intra­molecular hydrogen bond with the adjacent carbonyl O atom (Table 2 ▸). In the crystal packing, the mol­ecules form stacks, each of which consists of repeats of just one of the independent mol­ecules. The mol­ecules containing atom O1 lie tilted within an otherwise uniform column that runs parallel to the [100] direction. The mol­ecular plane is tilted by approximately 45° with respect to the stacking direction. Nonetheless, there are no significant π–π inter­actions, because the ring offsets resulting from the tilting preclude significant overlap of the ring systems. The mol­ecules containing atom O21 also stack parallel to the [100] direction in a similar 45°-tilted fashion, but the orientation of the tilted planes differs from that in the O1-containing stacks (Fig. 2 ▸); the normals to the mol­ecular planes in the two independent stacks point in different directions. Each type of stack runs parallel to another stack of the same kind related by a centre of inversion to give a centrosymmetric double-stack pair. As the planes of the mol­ecules in the two independent types of pairs of stacks are oriented differently, π–π inter­actions between the stacks are precluded and the stacks are not inter­twined with one another.

The Cambridge Structural Database (CSD, Version 2020.3.0 with May 2021 update; Groom *et al.*, 2016 ▸) contains data for six closely related flavones with hy­droxy or meth­oxy substituents at least at the 5-, 6-, 7-, 3′- and 4′-positions. The ring systems in four of these structures are planar, with perhaps a tendency towards a slight bowing along the axis of the three-ring system, similar to that observed in (**2**), as seen solely from visual inspection. These structures are 5,7,4′-trihy­droxy-6,3′,5′-tri­meth­oxy­flavone ethyl acetate solvate (Martinez-Vazquez *et al.*, 1993 ▸), 5,3′-dihy­droxy-6,7,4′-tri­meth­oxy­flavone (Parvez *et al.*, 2001 ▸), 5,7-dihy­droxy-6,3′,4′-tri­meth­oxy­flavone (Suleimenov *et al.*, 2005 ▸) and 5,7,3′-trihy­droxy-6,4′,5′-tri­meth­oxy­flavone (Adizov *et al.*, 2013 ▸; Turdybekov *et al.*, 2014 ▸). In the structure of 5,6,7,2′,3′,4′-hexa­meth­oxy­flavone (Butler *et al.*, 2018 ▸), the bowing within the fused rings appears to be more prominent. In the structure of 5,3′-dihy­droxy-6,7,2′,4′,5′-penta­meth­oxy­flavone (Al-Yahya *et al.*, 1987 ▸), the individual planes of the phenyl and fused rings are significantly tilted from one another, with a dihedral angle of 12.23 (14)°; this is the only example with four substituents on the phenyl ring (three meth­oxy and one hy­droxy).

**Figure 3:**
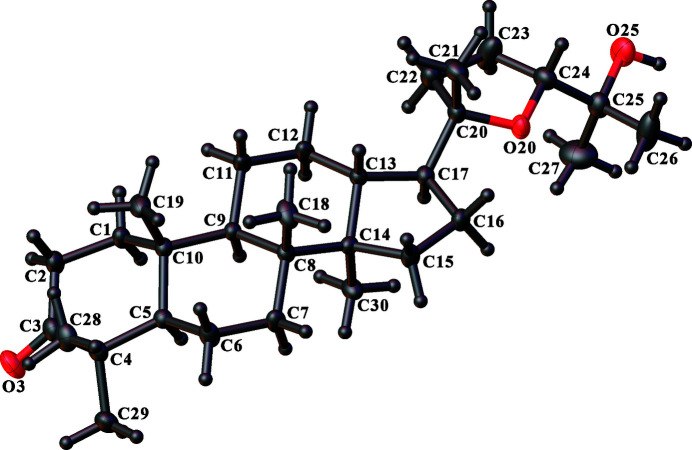
View of the mol­ecule of (**3**), showing the atom-labelling scheme. Displacement ellipsoids are drawn at the 50% probability level. H atoms are represented by spheres of arbitrary size.

The crystal structure of the triterpene (20*S*,24*R*)-ocotillone, (**3**), has one mol­ecule in the asymmetric unit (Fig. 3 ▸). In the chosen crystal, the com­pound is enanti­omerically pure and the absolute configuration of the mol­ecule was determined independently by the diffraction experiment; the value of the absolute structure parameter (Parsons *et al.*, 2013 ▸) was −0.07 (4). According to the numbering of the atoms used in the refinement model, the absolute configuration of the stereogenic C atoms of the mol­ecule is established as follows: 5*R*,8*R*,9*R*,10*R*,13*R*,14*R*,17*S*,20*S*,24*R*. The isolation and identification of 20*S*- and 20*R*-ocotillones have been reported on several occasions (Bisset *et al.*, 1966 ▸, 1967 ▸; Betancor *et al.*, 1983 ▸; Aalbersberg *et al.*, 1991 ▸). The isolation of the corresponding alcohol, ocotillol, appears to be mentioned for the first time by Warnhoff & Halls (1965 ▸). The absolute configuration of (20*S*,24*R*)-ocotillone was deduced from an X-ray crystal structure of a bromo­benzoyl derivative of the corresponding ocotillol (Yamauchi *et al.*, 1969 ▸). The crystal structure determination of (**3**) is the first time the absolute configuration has been confirmed crystallographically for the native (20*S*,24*R*)-ocotillone.

The core of the mol­ecule of (**3**) consists of five rings, including four fused rings, cyclo­hexane rings *A* (atoms C1–C5/C10), *B* (C5–C10) and *C* (C8/C9/C11–C14), and cyclo­pentane ring *D* (C13–C17), plus furan ring *E* (O18/C20–C24) attached to the fused rings at atom C17. An iso­propanol substituent is present at atom C24 of the furan ring. Thus, com­pound (**3**) is (5*R*,8*R*,9*R*,10*R*,13*R*,14*R*,17*S*)-17-[(2*S*,5*R*)-5-(2-hy­droxy­pro­pan-2-yl)-2-methyloxolan-2-yl]-4,4,8,10,14-penta­methyl-1,2,5,6,7,9,11,12,13,15,16,17-dodeca­hydrocyclo­penta­[*a*]phenanthren-3-one. Rings *A*, *B* and *C* adopt a chair conformation, with ring *A* being the most distorted because of the presence of the *sp* ^2^-hybridized keto C atom. The puckering parameters (Cremer & Pople, 1975 ▸) for ring *A* are θ = 15.82 (18)° and φ = 322.1 (7)° for the atom sequence C1—C2—C3—C4—C5—C10. For ring *B*, θ = 11.01 (15)° and φ = 17.2 (8)° for the atom sequence C5—C6—C7—C8—C9—C10 and for ring *C*, θ = 6.30 (15)° and φ = 8.5 (13)° for the atom sequence C8—C9—C11—C12—C13—C14. Ring *D* has a near-ideal half-chair conformation twisted on C13—C14 [φ_2_ = 197.8 (4)° for the atom sequence C13—C14—C15—C16—C17], while ring *E* has a slightly distorted envelope conformation with atom O20 as the envelope flap [φ_2_ = 188.6 (4)° for the atom sequence O20—C21—C22—C23—C24]. The *A*/*B*, *B*/*C* and *C*/*D* ring junctions are all *trans*-fused to each other along the C5—C10, C8—C9 and C13—C14 bonds, respectively. This brings the methyl groups at C8 and C10 into *cis* positions, while the methyl groups at C8 and C14 are *trans* to one another. The furan substituent at the cyclo­propane ring lies *trans* to the C14 methyl group.

**Figure 4:**
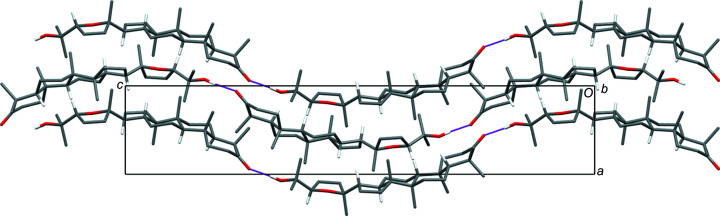
The crystal packing of (**3**), viewed down the *b* axis, showing the O—H⋯O hydrogen bonds (magenta dashed lines) linking the mol­ecules into wave-like chains. Most H atoms have been omitted for clarity.

Inter­molecular O—H⋯O hydrogen bonds involving the hy­droxy group and the ketone O atom link the mol­ecules into extended wave-like chains (Table 3 ▸ and Fig. 4 ▸), which run parallel to the [001] direction and can be described by a graph-set motif (Bernstein *et al.*, 1995 ▸) of *C*(16).

### Anthelmintic activity   

#### About the extracts   

**Figure 5:**
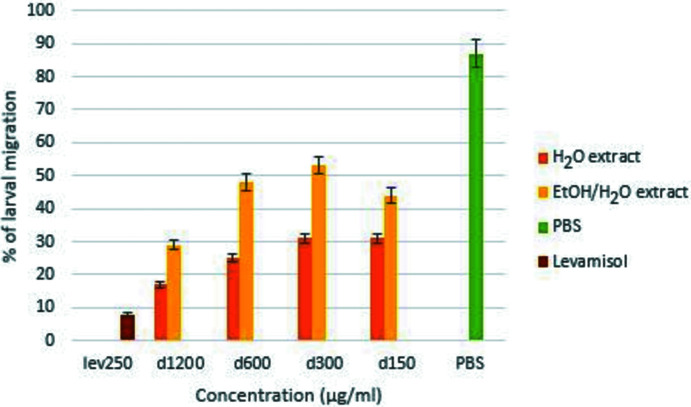
The effect on *H. contortus* larval migration caused by *C. glutinosum* extracts.

The crude extracts obtained by aqueous decoction and hydro-ethano­lic maceration, as well as the nine isolated com­pounds, were tested for their anthelmintic activity on the larvae and adult worms of *H. contortus*. The larval migration inhibition technique applied is based on the measurement of the migration rate of parasite larvae through a membrane after contact with the tested extract. At different doses, aqueous and hydro-ethanol extracts of *C. glutinosum* significantly inhibited *in vitro* larval migration of *H. contortus* (*p* < 0.001) (Fig. 5 ▸). This effect is independent of the dose and does not vary with the extraction solvent (*p* > 0.05). However, the aqueous extract appeared to be more effective than the hydro-ethano­lic extract (Fig. 5 ▸). Similarly, both extracts significantly reduced the motility of adult *H. contortus* worms (*p* < 0.001). Although the inhibition effect did not vary with dose and extraction solvent (*p* > 0.05), it did vary with time (*p* < 0.001) and, paradoxically, the hydro-ethano­lic extract appeared to inhibit adult worm motility more (Table 4 ▸). In order to know the chemical com­position of these two extracts for the identification of the active principle, the present work was continued with the hydro-ethano­lic extract and the com­pounds isolated therefrom were tested on *H. contortus* larvae and worms.

#### On the com­pounds   

**Figure 6:**
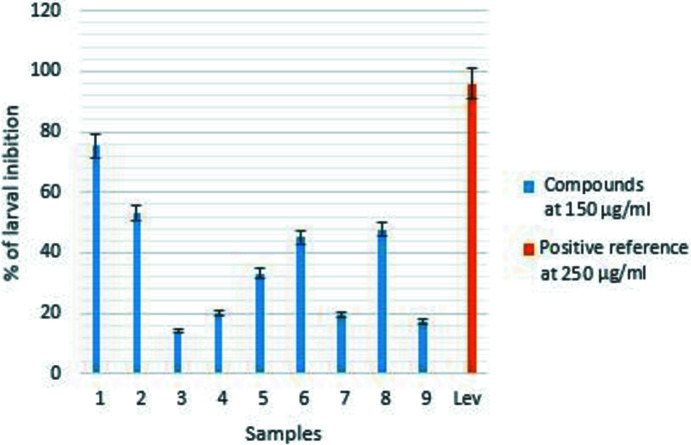
Inhibition of *H. contortus* larval migration by the com­pounds isolated from *C. glutinosum*.

*In vitro*, the effect of the com­pounds was evaluated on *H. contortus* larvae and adult worms. All the com­pounds inhibited the migration of *H. contortus* larvae (Fig. 6 ▸) and the three isolated flavonoids seem to present the best results with inhibition percentages of 75.37, 53.26 and 47.73%, respectively, for com­pounds (**1**), (**2**) and (**8**), although they are all less active than the reference drug levamisol (95.97%). For the adult worms observed every 3 h with a magnifying glass after their contact with the tested com­pounds, the total inhibition of their motility was observed with the positive reference control (levamisol) after just 3 h of exposure. This inhibition was total at 12 h with com­pounds (**1**), (**2**), (**4**), (**5**) and (**8**). On the other hand, in phosphate buffer solution (PBS), 75% of adult worms were still mobile after 18 h (Table 5 ▸). Statistical analysis showed that the com­pounds inhibited the larval migration and motility of *H. contortus* adult worms within the same time as levamisole, com­pared with the negative control (*p* < 0.001). On adult worms, the inhibitory effect varied with time (*p* < 0.001) and flavonoids; in particular, 5-de­methyl­sinensetin, (**1**), would be responsible for the known anthelmintic activity of the plant.

Indeed, the class of polyphenols is strongly suspected as being the active agent in the anthelmintic effect of plants (Ayers *et al.*, 2008 ▸). Condensed tannins are frequently re­ported as being responsible for such effects, for example, in the report by Hoste *et al.* (2018 ▸). Nonetheless, other reports do link anthelmintic properties to flavonoids (Paolini *et al.*, 2003 ▸; Barrau *et al.*, 2005 ▸). Given the results of the *in vivo* tests, the known anthelmintic activity of *C. glutinosum* appears to be related to the presence of the flavonoids isolated from this plant. Thus, following the report that *C. glutinosum* is an anthelmintic plant (Alowanou *et al.*, 2019 ▸), the present study has allowed the anthelmintic capacity of the different com­pounds isolated from this plant to be ranked and highlighted. It appears that these com­pounds, although less active than the positive reference control, have a larvicidal and vermicidal effect on *H. contortus*, with 5-de­methyl­sinensetin, (**1**), being the most active. The decrease in the migration of infesting larvae and the reduction of the motility of adult worms could disrupt their settlement in the mucosal wall of the digestive tract and thus ensure their progressive elimination from the infested animal (Dedehou *et al.*, 2014 ▸). These results could serve as a basis for a conformational analysis leading to the proposal of a new com­pound with a broader spectrum of activity than current commercially available anthelmintics.

## Conclusion   

The phytochemical investigation of the leaves of *C. glutinosum* led to the isolation of nine known com­pounds, which were characterized using spectroscopic analyses and by com­parison with literature data. The crystal structures of two com­pounds were described for the first time in the present work and four com­pounds have been isolated for the first time from the genus *Combretum*. The flavonoids isolated from the plant presented the best *in vitro* activity on *H. contortus*. The results of this study could be verified *in vivo* on sheep in order to gain further insight into and enhance the status of this plant.

## Supplementary Material

Crystal structure: contains datablock(s) 2, 3, global. DOI: 10.1107/S2053229621007841/ov3154sup1.cif

Structure factors: contains datablock(s) 2. DOI: 10.1107/S2053229621007841/ov31542sup2.hkl

Supporting information file. DOI: 10.1107/S2053229621007841/ov31542sup4.mol

Structure factors: contains datablock(s) 3. DOI: 10.1107/S2053229621007841/ov31543sup3.hkl

Supporting information file. DOI: 10.1107/S2053229621007841/ov31542sup5.cml

Supporting information file. DOI: 10.1107/S2053229621007841/ov31543sup6.cml

CCDC references: 2100535, 2100534

## Figures and Tables

**Table 1:** Experimental details For both structures: *Z* = 4. Experiments were carried out at 160 K with Cu *K*α radiation. H atoms were treated by a mixture of independent and constrained refinement. The absorption correction was numerical based on Gaussian integration over a multifaceted crystal model (Coppens *et al.*, 1965 ▸) plus empirical (using intensity measurements) using spherical harmonics (*CrysAlis PRO*; Rigaku Oxford Diffraction, 2021 ▸).

	(**2**)	(**3**)
Crystal data		
Chemical formula	C_20_H_20_O_8_	C_30_H_50_O_3_
*M*_r_	388.36	458.70
Crystal system, space group	Triclinic, *P*\overline{1}	Orthorhombic, *P*2_1_2_1_2_1_
*a*, *b*, *c* (Å)	4.97902 (15), 18.5654 (5), 19.1368 (3)	6.37386 (6), 12.10746 (11), 33.8928 (3)
α, β, γ (°)	89.5065 (18), 84.322 (2), 89.375 (2)	90, 90, 90
*V* (Å^3^)	1760.12 (8)	2615.55 (4)
μ (mm^−1^)	0.96	0.56
Crystal size (mm)	0.17 × 0.03 × 0.01	0.24 × 0.19 × 0.05

Data collection		
Diffractometer	Rigaku Oxford Diffraction XtaLAB Synergy dual radiation	Oxford Diffraction SuperNova dual radiation
*T*_min_, *T*_max_	0.694, 1.000	0.614, 1.000
No. of measured, independent and observed [*I* > 2σ(*I*)] reflections	34916, 6662, 5215	26797, 5424, 5324
*R*_int_	0.060	0.018
(sin θ/λ)_max_ (Å^−1^)	0.610	0.630

Refinement		
*R*[*F*^2^ > 2σ(*F* ^2^)], *wR*(*F* ^2^), *S*	0.047, 0.113, 1.05	0.032, 0.089, 1.03
No. of reflections	6661	5424
No. of parameters	524	310
Δρ_max_, Δρ_min_ (e Å^−3^)	0.44, −0.23	0.23, −0.14
Absolute structure	–	Flack *x* determined using 2226 quotients [(*I* ^+^) − (*I* ^−^)]/[(*I* ^+^) + (*I* ^−^)] (Parsons *et al.*, 2013 ▸)
Absolute structure parameter	–	−0.07 (4)

Computer programs: *CrysAlis PRO* (Rigaku Oxford Diffraction, 2021 ▸), *SHELXT2018* (Sheldrick, 2015*a* ▸), *OLEX2* (Dolomanov *et al.*, 2009 ▸), *Mercury* (Macrae *et al.*, 2020 ▸), *SHELXL2018* (Sheldrick, 2015*b* ▸) and *PLATON* (Spek, 2020 ▸).

**Table 2:** Hydrogen-bond geometry (Å, °) for (**2**)

*D*—H⋯*A*	*D*—H	H⋯*A*	*D*⋯*A*	*D*—H⋯*A*
O5—H5⋯O4	0.89 (3)	1.75 (3)	2.595 (2)	159 (3)
O25—H25⋯O24	0.96 (3)	1.68 (3)	2.591 (2)	155 (3)

**Table 3:** Hydrogen-bond geometry (Å, °) for (**3**)

*D*—H⋯*A*	*D*—H	H⋯*A*	*D*⋯*A*	*D*—H⋯*A*
O25—H25⋯O3^i^	0.90 (3)	2.03 (3)	2.9325 (18)	172 (3)

Symmetry code: (i) -x+{\script{1\over 2}}, -y+1, z-{\script{1\over 2}}.

**Table 4:** The motility (%) of adult *H. contortus* worms in the presence of different concentrations of *C. glutinosum* extracts and reference control media

Sample	Concentration	Time				
	(dose, µg ml^−1^)	6 h	12 h	18 h	24 h	30 h
PBS	d0	100	100	66.7	33.3	0
Levamisol	d500	50	50	0	0	0
	d250	66.7	0	0	0	0
	d125	0	0	0	0	0
Aqueous	d2400	100	25	0	0	0
extract	d1200	100	75	25	0	0
	d600	100	100	0	0	0
	d300	100	66.7	0	0	0
	d150	100	33.3	25	0	0
	d75	100	75	50	0	0
Ethanol/water	d2400	100	0	0	0	0
extract	d1200	100	33.3	0	0	0
	d600	100	75	25	0	0
	d300	66.67	66.7	0	0	0
	d150	100	50	0	0	0
	d75	100	33.3	0	0	0

**Table 5:** The motility (%) of adult *H. contortus* worms in the presence of the isolated com­pounds (150 µg ml^−1^), as determined by an adult worm motility inhibition assay (AMIA)

Compound	Time					
	3 h	6 h	9 h	12 h	15 h	18 h
5-De­methyl­sinensetin, (**1**)	100	50	0	0	0	0
Umuhengerin, (**2**)	100	75	0	0	0	0
Ocotillone, (**3**)	100	100	25	25	0	0
Lupeol, (**4**)	100	100	0	0	0	0
β-Sitosterol, (**5**)	100	100	0	0	0	0
Oleanolic acid, (**6**)	100	100	25	0	0	0
Betulinic acid, (**7**)	100	100	25	25	0	0
Corymbosin, (**8**)	100	100	0	0	0	0
β-Sitosterol glucoside, (**9**)	100	50	25	25	0	0
Levamisol	25	0	0	0	0	0
PBS	100	100	100	100	100	75
